# Association of Electronic Cigarette Use With Subsequent Initiation of Tobacco Cigarettes in US Youths

**DOI:** 10.1001/jamanetworkopen.2018.7794

**Published:** 2019-02-01

**Authors:** Kaitlyn M. Berry, Jessica L. Fetterman, Emelia J. Benjamin, Aruni Bhatnagar, Jessica L. Barrington-Trimis, Adam M. Leventhal, Andrew Stokes

**Affiliations:** 1 Department of Global Health, Boston University School of Public Health, Boston, Massachusetts; 2 Department of Medicine, Boston University School of Medicine, Boston, Massachusetts; 3 Department of Epidemiology, Boston University School of Public Health, Boston, Massachusetts; 4 Department of Medicine, University of Louisville, Louisville, Kentucky; 5 Department of Preventive Medicine, Keck School of Medicine, University of Southern California, Los Angeles

## Abstract

**Importance:**

The use of electronic cigarettes (e-cigarettes) and other noncigarette tobacco products may increase the odds of cigarette initiation, even among low-risk youths.

**Objective:**

To evaluate the associations of prior e-cigarette use and other tobacco product use with subsequent cigarette initiation within 2 years of follow-up.

**Design, Setting, and Participants:**

In this prospective cohort study, data from waves 1 through 3 of the Population Assessment of Tobacco and Health Study (2013-2016) were used to assess youths aged 12 to 15 years who had never used cigarettes, e-cigarettes, or other tobacco products at wave 1. This was a nationally representative study of the US population. Data analysis was conducted in 2018.

**Exposures:**

First noncigarette tobacco product used (none, e-cigarette, or other tobacco product) between wave 1 and wave 3.

**Main Outcomes and Measures:**

Ever cigarette use and current cigarette use at wave 3.

**Results:**

In the sample (N = 6123), respondents were 49.5% female; 54.1% non-Hispanic, white; and the mean (SD) age was 13.4 (1.2) years. Of these, 8.6% reported e-cigarettes as their first tobacco product, while 5.0% reported using another noncigarette product first; 3.3% reported using cigarettes first. Cigarette use at wave 3 was higher among prior e-cigarette users (20.5%) compared with youths with no prior tobacco use (3.8%). Prior e-cigarette use was associated with more than 4 times the odds of ever cigarette use (odds ratio, 4.09; 95% CI, 2.97-5.63) and nearly 3 times the odds of current cigarette use (odds ratio, 2.75; 95% CI, 1.60-4.73) compared with no prior tobacco use. Prior use of other tobacco products was similarly associated with subsequent ever cigarette use (OR, 3.84; 95% CI, 2.63-5.63) and current cigarette use (OR, 3.43; 95% CI, 1.88-6.26) compared with no prior tobacco use. The association of prior e-cigarette use with cigarette initiation was stronger among low-risk youths (OR, 8.57; 95% CI, 3.87-18.97), a pattern not seen for prior other product use. Over the 2 years between 2013 and 2014 and 2015 and 2016, 21.8% of new cigarette ever use (178 850 youths) and 15.3% of current cigarette use (43 446 youths) among US youths aged 12 to 15 years may be attributable to prior e-cigarette use.

**Conclusions and Relevance:**

This study’s findings support the notion that e-cigarette use is associated with increased risk for cigarette initiation and use, particularly among low-risk youths. At the population level, the use of e-cigarettes may be a contributor to the initiation of cigarette smoking among youths.

## Key Points

**Question:**

Is electronic cigarette (e-cigarette) use among tobacco-naive youths associated with subsequent risk of cigarette initiation?

**Findings:**

In this cohort study using data from the Population Assessment of Tobacco and Health Study (2013-2016), youths whose first tobacco product was an e-cigarette were more likely to initiate cigarettes over 2 years of follow-up. At the population level, approximately 180 000 new ever smokers and 45 000 current smokers in the United States over 2 years may have started smoking combustible cigarettes after initiating e-cigarette use.

**Meaning:**

Tobacco-naive youths who initiate e-cigarettes may be at greater risk of subsequently initiating cigarette smoking.

## Teaser

This cohort study evaluates whether prior use of electronic cigarettes (e-cigarettes) and other tobacco products by US youths is associated with subsequent initation of cigarette smoking.

## Introduction

In recent years, the proportion of US youths using electronic cigarettes (e-cigarettes), which are considered tobacco products by the US Food and Drug Administration,^1^ has increased rapidly.^2^ Past 30-day use of e-cigarettes among high school students increased from 1.5% in 2011 to 20.8% in 2018, with a 78% increase (from 11.7% to 20.8%) from 2017 to 2018.^3^ These trends have raised concerns that e-cigarettes may lead to the renormalization of tobacco and the initiation of new generations of youths into cigarette smoking,^4,5^ thus reversing decades of progress in reducing the burden of tobacco-related disease and mortality.^6^

Evidence from studies on diverse populations suggests that e-cigarette use may be associated with initiation of combustible cigarette smoking among youths.^7,8,9,10,11,12,13,14,15,16,17^ A recent meta-analysis^18^ found that youth and young adult e-cigarette users had more than 3 times the odds of subsequent cigarette initiation and more than 4 times the odds of past 30-day cigarette smoking. Based on this evidence, the 2018 National Academies of Sciences, Engineering, and Medicine Report concluded, “There is substantial evidence that e-cigarette use increases risk of ever using combustible tobacco cigarettes among youth and young adults.”^19^

Although prior studies have shown a significant association between e-cigarette use and subsequent cigarette initiation, they may be subject to methodological limitations. First, the commonly used restriction of samples to baseline never cigarette users^18^ may introduce selection bias by retaining only e-cigarette users who did not already progress to cigarette smoking. Second, the reference group of e-cigarette nonusers at baseline may be subject to exposure misclassification by including youths who begin using e-cigarettes after that point but prior to cigarette outcome assessment. Third, by examining an exposure that is already established at baseline and applying contemporaneous adjustment for alcohol and marijuana use,^13,17^ prior studies risk adjustment for variables that fall in the pathway between exposure and outcome.

As with e-cigarettes, use of other noncigarette tobacco products may increase risk of cigarette initiation. Previous longitudinal studies have reported that the use of smokeless tobacco^20,21,22^ or hookah^22,23^ is associated with subsequent cigarette initiation. With some notable exceptions, few studies have simultaneously assessed the associations of both e-cigarettes and other noncigarette tobacco products with future smoking. Therefore, additional evidence comparing the association between e-cigarette use and cigarette initiation with the association between other tobacco use and smoking is needed to contextualize the individual-level and population-level impact of e-cigarettes on smoking initiation.

Although strong individual-level associations have been reported in the existing literature, the aggregate effect of e-cigarette use on population-level cigarette initiation is uncertain because the increase in e-cigarette use has been accompanied by a decline in smoking prevalence.^19,24^ Still, the total number of youths initiating combustible cigarette use through e-cigarettes may represent a substantial, and preventable, public health burden. Hence, additional empirical estimates derived from internally consistent data sources are needed to assess the population-level effect of e-cigarettes, and other tobacco products, on combustible cigarette initiation.

We analyzed data from 3 waves of a large, nationally representative sample of the United States, the Population Assessment of Tobacco and Health (PATH) Study^25^ (2013-2016), to evaluate the associations of prior e-cigarette and other noncigarette tobacco product use with subsequent cigarette initiation over approximately 2 years. Using a study design based on the first ever tobacco product used, we addressed the potential for biases in prior longitudinal studies investigating this research question. Additionally, we evaluated the consistency of associations across groups defined by risk-taking propensities and examined the implications of our estimates for the overall proportion of US youths likely to initiate cigarette smoking as a result of e-cigarette use or use of another noncigarette tobacco product.

## Methods

### Study Sample

The PATH Study is a large, nationally representative cohort administered by the National Institutes of Health and the US Food and Drug Administration.^25,26^ The study, which collects extensive information on tobacco use, used a 4-stage, stratified, probability sample design. Data for wave 1 were collected between September 2013 and December 2014 and included 13 651 youths (aged 12-17 years). Data for wave 2 were collected from 2013 and 2015, and data for wave 3 were collected between 2015 and 2016.^25^ Overall, 11 046 youths (80.9%) were retained between wave 1 and wave 3, including those who aged into the adult sample.

Our analysis relied on deidentified data and was therefore exempted from review by the Boston University Medical Center institutional review board. We followed the Strengthening the Reporting of Observational Studies in Epidemiology (STROBE) reporting guideline for observational studies to guide our reporting. Our analysis was restricted to youths aged 12 to 15 years who had never used any tobacco product at wave 1. The youths aged 12 to 15 years group was chosen so that the sample would remain in the PATH Study youth sample at all 3 waves, allowing us to take advantage of the more detailed youth questionnaire to establish temporal ordering of product use. We excluded participants missing information needed to determine exposure or outcome status, yielding a final analytic sample of 6123 youths (eFigure in the Supplement).

### Prior Tobacco Product Use

We assessed tobacco product use through a combination of questions on current and prior behavior from the wave 2 and wave 3 questionnaires. We determined the temporal ordering of product use through the question “You mentioned that you started using (list of all products used) in the last 12 months. Which type of tobacco did you try first?” Youths who remained tobacco naive at wave 3 were classified as having no prior tobacco use. We classified youths who reported e-cigarettes as their first product as prior e-cigarette users, while youths who reported using another noncigarette product (eg, cigar, cigarillo, filtered cigar, pipe, hookah, smokeless tobacco, snus, dissolvable tobacco, bidi, or kretek) first were considered prior users of other products. We assigned youths who reported cigarette use only to the no prior tobacco use reference group as cigarette use was the outcome of interest. Our method of exposure classification is illustrated in the Figure.

**Figure.**
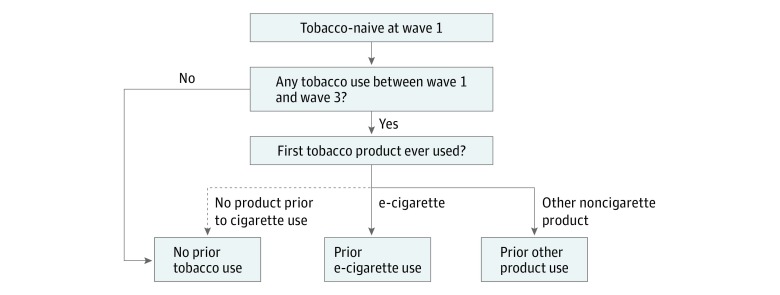
Exposure Classification Exposure to electronic cigarettes (e-cigarettes) and other tobacco products prior to cigarette use was assessed based on first tobacco product ever used. Youths who reported e-cigarettes as their only or first product between wave 1 and wave 3 were classified as prior e-cigarette users, while youths who reported using another noncigarette tobacco product (cigar, cigarillo, filtered cigar, pipe, hookah, smokeless tobacco, snus, dissolvable tobacco, bidi, or kretek) first were considered prior other product users. Youths who reported cigarette use without prior use of e-cigarettes or other tobacco products were classified into the no prior tobacco use reference group as cigarette use was the outcome of interest.

### Cigarette Use

We considered youths to be ever users of cigarettes if they responded yes to the question “Have you ever tried cigarette smoking, even 1 or 2 puffs?” at wave 3. We defined current cigarette use as use of cigarettes in the 30 days prior to wave 3.

### Risk Group Measures

Ever use of alcohol, marijuana, and prescription drug abuse (ie, use of methylphenidate [Ritalin], Adderall, painkillers, sedatives, and tranquilizers without a prescription) was assessed at wave 1. Respondents were also asked about their agreement with sensation-seeking statements including affinity for frightening things, new and exciting things, and unpredictable friends.^27^ Cigarette susceptibility was assessed via 3 questions: (1) Have you ever been curious about smoking a cigarette? (2) Do you think you will smoke a cigarette in the next year? and (3) If one of your best friends were to offer you a cigarette, would you smoke it?

These 9 variables were used to categorize youths into an intermediate- and/or high-risk group and a low-risk group (eTable 1 in the Supplement). Youths reporting any risky behaviors, any sensation-seeking personality traits, or any cigarette susceptibility were considered intermediate and/or high risk. Youths who did not exhibit any risk components were considered low risk.

### Other Baseline Measures

During wave 1 of the PATH Study, data on age, sex, race and ethnicity (non-Hispanic, white; non-Hispanic, black; non-Hispanic, other; Hispanic), parental education (<college degree or ≥college degree), and urban or rural residence were collected. During this wave, youths were also asked whether they lived with a tobacco user (yes or no), the frequency of noticing warnings on cigarette packages (never, rarely, sometimes, often, very often), and the ability to recall a favorite tobacco advertisement (has favorite tobacco advertisement, no favorite tobacco advertisement).

### Statistical Analysis

We used multivariable logistic regression analyses to evaluate the odds of ever and current cigarette use at wave 3 as a function of prior tobacco product use (prior use of e-cigarettes, prior use of other products, or no prior tobacco use). We adjusted each model for wave 1 characteristics including sex, race and ethnicity, parental education, urban residence, living with a tobacco user, frequency of noticing health warnings on cigarette packages, and ability to recall a favorite tobacco advertisement.^28^ We also adjusted the models for wave 1 risk-taking behaviors, sensation-seeking personality traits, and cigarette susceptibility. We calculated predicted probabilities of ever and current cigarette use through marginal standardization using the coefficients produced by regression models.

In addition, we stratified the regressions of ever and current cigarette use at wave 3 by baseline risk group (intermediate- and/or high-risk vs low-risk). We adjusted each stratified regression model for the same demographic factors and established determinants of cigarette use as primary models. We additionally adjusted the intermediate- and/or high-risk strata for individual components of risk-taking behaviors, sensation-seeking personality traits, and cigarette susceptibility to account for remaining differences in baseline risk.

We used the PATH Study–generated imputed variables for age, sex, and race and ethnicity. We used multiple imputation by chained equations (5 imputations) to account for missing data in independent variables other than exposure.^29^ To test the sensitivity of the findings to missing data, we refit all regression models prior to imputation using a subset of the data with no missing information on any covariate.

We estimated the percentage of cigarette use that could be averted under the hypothetical scenario in which prior e-cigarette users had not taken up e-cigarettes using our multivariable-adjusted risk estimates to calculate population-attributable fractions (PAFs).^30,31^ Because our outcomes were rare (6.1% for ever cigarette use and 2.1% for current cigarette use), our odds ratios (ORs) approximate risk ratios. We used the PATH Study sample weights to estimate the total number of new youth cigarette initiators in the United States at wave 3 and multiplied this total by the PAF to determine the total number of new youth cigarette smokers attributable to prior e-cigarette use over the 2-year period. We repeated this process for prior other product use to determine the proportion of subsequent cigarette use attributable to using another noncigarette tobacco product first. In a sensitivity analysis, we estimated risk ratios from our ORs using the correction method proposed by Zhang and Yu^32^ and repeated our PAF calculations using the corrected risk ratios.

Finally, we reversed our exposure and outcome to investigate the association of cigarette use with subsequent e-cigarette use, using multivariable logistic regression with the same set of covariates. In this analysis, youths whose first tobacco product was a cigarette were considered prior cigarette users.

We analyzed the data using Stata, version 12 (StataCorp). To adjust for unequal probabilities of selection and nonresponse,^26^ we used sample weights from wave 3 of the PATH Study derived by the PATH team. We estimated variances using Taylor series linearization with the survey routine and considered 2-sided *P* < .05 statistically significant.

## Results

Our study included 6123 youths who were tobacco naive at wave 1. The participants were 49.5% female; 54.1% non-Hispanic, white; and the mean (SD) age was 13.4 (1.2) years (Table 1). By wave 3, 8.6% of the sample reported e-cigarettes and 5.0% reported another noncigarette product as their first tobacco product, whereas 3.3% reported using cigarettes first.

**Table 1.** Characteristics of 6123 Tobacco-Naive Youths at Wave 1, Population Assessment of Tobacco and Health Study, 2013-2014

Wave 1	%^a^
Female	49.5
Age, y	
12	27.3
13	26.5
14	25.0
15	21.3
Race/ethnicity	
Non-Hispanic, white	54.1
Non-Hispanic, black	13.9
Hispanic	22.8
Non-Hispanic, other	9.2
Parent completed college or higher	35.9
Urban residence	80.4
Lives with tobacco user	28.9
Has favorite tobacco advertisement	6.5
Frequency of noticing tobacco warnings	
Never	55.5
Rarely	16.0
Sometimes	12.1
Often	8.4
Very often	8.0
Ever used alcohol	23.8
Ever used marijuana	1.2
Ever abused prescription drugs	6.3
Likes frightening things^b^	14.1
Likes new and exciting experiences^b^	15.9
Prefers wild and unpredictable friends^b^	27.4
Plans to smoke in next year^c^	10.0
Ever been curious about cigarettes^c^	21.3
Would smoke if offered cigarette by friend^c^	12.1
Risk group^d^	
Low risk	42.1
Intermediate and/or high risk	57.9
Prior tobacco product use^e^	
None	86.4
Prior e-cigarette use	8.6
Prior other product use	5.0
Cigarette use at wave 3	
Ever	6.1
Current	2.1

Abbreviation: e-cigarette, electronic cigarette.

^a^ Percentages were weighted using wave 3 sample weights. Guidelines for the Restricted Use Files of the Population Assessment of Tobacco and Health Study prohibit the reporting of cell counts.

^b^ For sensation-seeking personality trait questions, estimates indicate the percentages of respondents who strongly agreed or agreed with the statements.

^c^ For cigarette-susceptibility questions, responses of not at all or definitely not were considered nonsusceptible. All other responses were considered susceptible.

^d^ Youths reporting any of the following were considered intermediate or high risk: ever alcohol use, ever marijuana use, prescription drug abuse, enjoying frightening things, liking new and exciting experiences, preferring unpredictable friends, willingness to smoke in next year, curiosity about cigarettes, or susceptibility to cigarette peer pressure from friends. Youths who did not exhibit any risk behaviors, sensation-seeking personality traits, or cigarette susceptibility were considered low risk.

^e^ Youths were considered to have prior e-cigarette use if they started using e-cigarettes between wave 1 and wave 3 and their e-cigarette use preceded use of any other tobacco product. They were considered to have other product use if another noncigarette tobacco product was their first product.

Ever cigarette use at wave 3 (6.1% overall) was higher among prior users of e-cigarettes (20.5%) and prior users of other products (21.1%) compared with youths with no prior tobacco use (3.8%). Similarly, past 30-day use of cigarettes at wave 3 (2.1% of total sample) was highest among prior users of another tobacco product (8.2%), followed by prior users of e-cigarettes (5.9%), and those with no prior tobacco use (1.4%).

Prior e-cigarette users had 4.09 (95% CI, 2.97-5.63) times the odds of ever cigarette use compared with youths with no prior tobacco use (Table 2), while prior other product users had 3.84 (95% CI, 2.63-5.63) times the odds of ever cigarette use. Additionally, the odds of current cigarette use at wave 3 were higher among prior e-cigarette users (OR, 2.75; 95% CI, 1.60-4.73) and prior other product users (OR, 3.43; 95% CI, 1.88-6.26) compared with youths with no prior tobacco use. Sensitivity analyses restricted to youths without missing covariate information yielded similar findings (eTable 2 in the Supplement).

**Table 2.** Adjusted Probabilities and Odds of Cigarette Use at Wave 3 by Prior Tobacco Product Use Among 6123 Youths Aged 12 to 15 Years, Population Assessment of Tobacco and Health Study, 2013-2016

Prior Tobacco Product Use^a^	Ever Cigarette Use			Current Cigarette Use		
	Adjusted OR (95% CI)^b^	*P* Value	Adjusted, %^c^	Adjusted OR (95% CI)^b^	*P* Value	Adjusted, %^c^
None	1 [Reference]	NA	4.3	1 [Reference]	NA	1.5
Prior e-cigarette use	4.09 (2.97-5.63)	<.001	13.8	2.75 (1.60-4.73)	<.001	4.0
Prior other product use	3.84 (2.63-5.63)	<.001	13.2	3.43 (1.88-6.26)	<.001	4.8

Abbreviations: e-cigarette, electronic cigarette; NA, not applicable; OR, odds ratio.

^a^ Youths were considered to have prior e-cigarette use if they started using e-cigarettes between wave 1 and wave 3 and their e-cigarette use preceded use of any other tobacco product. They were considered to have other product use if another noncigarette tobacco product was their first product.

^b^ Regression models and resulting probabilities were sample weighted and adjusted for sex, age, race and ethnicity, parental education, urban or rural residence, living with a tobacco user, noticing tobacco warnings, tobacco advertisement receptivity, ever alcohol use, ever marijuana use, prescription drug abuse, enjoying frightening things, liking new and exciting experiences, preferring unpredictable friends, willingness to smoke in next year, curiosity about cigarettes, and susceptibility to cigarette peer pressure from friends. All covariates were assessed at wave 1.

^c^ Predicted probabilities were calculated via marginal standardization using coefficients estimated from regression models.

When we stratified by wave 1 risk group (Table 3), we found that the association between prior e-cigarette use and subsequent ever cigarette use was stronger among low-risk youths (OR, 8.57; 95% CI, 3.87-18.97) than among intermediate- and/or high-risk youths (OR, 3.51; 95%, 2.52-4.89) (*P* for interaction = .02). However, those in the intermediate- and/or high-risk categories and low-risk categories using other tobacco products had similar odds of ever cigarette use (intermediate- and/or high-risk: OR, 3.71; 95% CI, 2.48-5.54 and low-risk: OR, 4.03; 95% CI, 1.36-11.99; *P* for interaction = .69). Using the coefficients from the multivariable logistic regression models to calculate predicted probabilities of cigarette use, prior e-cigarette users considered low risk had similar adjusted probability of becoming ever cigarette users (OR, 9.9%; 95% CI, 3.9%-15.9%) compared with intermediate- and/or high-risk youths with no prior tobacco use (OR, 6.5%; 95% CI, 5.4%-7.5%). We saw similar patterns, with stronger associations for prior e-cigarette users in the low-risk group, in the stratified regression of current cigarette use. Additionally, sensitivity analyses restricted to youths without missing data yielded similar findings (eTable 3 in the Supplement).

**Table 3.** Adjusted Probabilities and Odds of Cigarette Use at Wave 3 by Prior Tobacco Product Use Among 6123 Youths Aged 12 to 15 Years, Stratified by Risk Group, Population Assessment of Tobacco and Health Study, 2013-2016

Cigarette Use	Intermediate- and/or High-Risk Group (n = 3548)^a^			Low-Risk Group (n = 2575)^a^			*P* Value for Interaction
	Adjusted OR (95% CI)^b^	*P* Value	Adjusted, %^c^	Adjusted OR (95% CI)^b^	*P* Value	Adjusted, %^c^	
Ever cigarette use							
Prior tobacco product use^d^							
None	1 [Reference]	NA	6.5	1 [Reference]	NA	1.3	
Prior e-cigarette use	3.51 (2.52-4.89)	<.001	18.0	8.57 (3.87-18.97)	<.001	9.9	.02^e^
Prior other product use	3.71 (2.48-5.54)	<.001	18.7	4.03 (1.36-11.99)	.01	5.0	.69^f^
Current cigarette use							
Prior tobacco product use^d^							
None	1 [Reference]	NA	2.3	1 [Reference]	NA	0.5	
Prior e-cigarette use	2.16 (1.23-3.79)	.01	4.7	10.36 (3.11-34.54)	<.001	4.9	.03^e^
Prior other product use	3.31 (1.77-6.19)	<.001	6.8	3.41 (0.56-20.67)	.18	1.8	.66^f^

Abbreviations: e-cigarette, electronic cigarette; NA, not applicable; OR, odds ratio.

^a^ Youths who reported ever alcohol use, ever marijuana use, prescription drug abuse, agreed with any of 3 statements on sensation seeking, or showed any susceptibility to any of the 3 questions on cigarette susceptibility were considered intermediate and/or high risk. Youths who had never used alcohol, never used marijuana, never abused prescription drugs, disagreed with all 3 sensation seeking statements, and showed no cigarette susceptibility were considered low risk.

^b^ Regression models and resulting probabilities were sample weighted and adjusted for sex, age, race and ethnicity, parental education, urban or rural residence, living with a tobacco user, noticing tobacco warnings, and tobacco advertisement receptivity. The intermediate- and/or high-risk strata was also adjusted for all 9 individual measures of risk (ever alcohol use, ever marijuana use, prescription drug abuse, enjoying frightening things, liking new and exciting experiences, preferring unpredictable friends, willingness to smoke in next year, curiosity about cigarettes, and susceptibility to cigarette peer pressure from friends). All covariates were assessed at wave 1.

^c^ Predicted probabilities were calculated via marginal standardization using coefficients estimated from regression models.

^d^ Youths were considered to have prior e-cigarette use if they started using e-cigarettes between wave 1 and wave 3 and their e-cigarette use preceded use of any other tobacco product. They were considered to have other product use if another noncigarette tobacco product was their first product.

^e^ *P* value for interaction between e-cigarette exposure and being in the intermediate- and/or high-risk group.

^f^ *P* value for interaction between other product exposure and being in the intermediate- and/or high-risk group.

At the population level, our analysis indicated the fraction of ever cigarette use attributable to prior e-cigarette use was 21.8%, whereas the fraction attributable to prior other product use was 12.8%. Extrapolating estimates from our sample to the population of US youths aged 12 to 15 years, an estimated 820 414 youths had new ever use of cigarettes over the 2-year period, suggesting there might have been 178 850 fewer cigarette initiators if there was no uptake of e-cigarettes (Table 4). The fraction of new current cigarette use at wave 3 attributable to prior e-cigarette use was 15.3%, suggesting 43 446 current cigarette users (of a total of 283 964) might not have become smokers without e-cigarettes. The fraction of new current cigarette use attributable to initiating tobacco use with another noncigarette tobacco product was similar at 13.7%. The sensitivity analysis using corrected risk ratios in PAF calculations yielded comparable estimates (eTable 4 in the Supplement).

**Table 4.** Population-Level Proportion of Cigarette Use Attributable to Prior Use of e-Cigarettes and Other Tobacco Products, Population Assessment of Tobacco and Health Study, 2013-2016

Prior Use	Ever Cigarette Use			Current Cigarette Use		
	PAF, %	Total New Users Over 2 y, No.	Attributable Users, No.	PAF, %	Total New Users Over 2 y, No.	Attributable Users, No.
Prior e-cigarette use	21.8^a^	820 414	178 850^b^	15.3^c^	283 964	43 446^d^
Prior other product use	12.8^e^	820 414	105 013^f^	13.7^g^	283 964	38 903^h^
Overall	34.6	820 414	283 863	29.0	283 964	82 349

Abbreviations: e-cigarette, electronic cigarette; OR, odds ratio; PAF, population-attributable fractions.

^a^ PAF = [proportion of ever cigarette users with prior e-cigarette use (OR for prior e-cigarette use – 1)] / (OR for prior e-cigarette use) = (0.288[4.09 − 1])/(4.09) = 21.8%.

^b^ Attributable users = (PAF)(total new users) = (0.218)(820 414) = 178 850.

^c^ PAF = [proportion of current cigarette users with prior e-cigarette use (OR for prior e-cigarette use – 1)] / (OR for prior e-cigarette use) = [0.241(2.75 − 1)]/(2.75) = 15.3%.

^d^ Attributable users = (PAF)(total new users) = (0.153)(283 964) = 43 446.

^e^ PAF = [proportion of ever cigarette users with prior other product use (OR for prior other product use – 1)] / (OR for prior other product use) = [0.173(3.84 − 1)]/(3.84) = 12.8%.

^f^ Attributable users = (PAF)(total new users) = (0.128)(820 414) = 105 013.

^g^ PAF = [proportion of current cigarette users with prior other product use (OR for prior other product use – 1)] / (OR for prior other product use) = [0.194(3.43 − 1)]/(3.43) = 13.7%.

^h^ Attributable users = (PAF)(total new users) = (0.137)(283 964) = 38 903.

In the reverse analysis, youths reporting prior cigarette use had an odds ratio of 3.51 (95% CI, 2.40-5.14) for ever using of e-cigarettes compared with youths with no prior tobacco use (eTable 5 in the Supplement). The association between prior use of other tobacco products and subsequent ever e-cigarette use was not as strong (OR, 1.81; 95% CI, 1.28-2.54). Similar results were seen in the analysis of current e-cigarette use.

## Discussion

In this nationally representative study of US youths, we found that using e-cigarettes as one’s first tobacco product was associated with more than 4 times the odds of ever cigarette use and nearly 3 times the odds of current cigarette use over 2 years of follow-up. We also found that prior other product use was associated with similar odds of subsequent cigarette initiation. In stratified analyses, we found that the association of prior e-cigarette use and subsequent cigarette initiation were especially pronounced in low-risk youths, a pattern not seen for prior use of other products. Finally, we estimated that 21.8% of new ever cigarette use and 15.3% of current cigarette use in the US youth population may be attributable to initiating tobacco products through e-cigarette use. Comparatively, we estimate 12.8% of cigarette initiation and 13.7% of current use is attributable to the prior use of other noncigarette products. These estimates suggest that the proportion of smoking attributable to e-cigarettes may be larger than the proportion attributable to all other products combined.

We used methodological refinements associated with defining exposure based on a youth’s first tobacco product; nevertheless, our results are consistent with prior work suggesting that the use of e-cigarettes^18,19^ and other tobacco products^15,22^ is associated with increased risk of cigarette initiation. Prior studies have reported stronger associations among adolescents with no baseline intent to smoke^7^ or lower risk-taking behaviors.^33^ Similarly, we found that the association of prior e-cigarette use with subsequent cigarette use was especially pronounced in low-risk youths when we stratified our analysis by baseline risk-taking behaviors and traits. This observation suggests that propensity for risk^34,35^ is unlikely to be the sole explanation for the association between e-cigarette use and cigarette initiation. The same pattern of heightened risk among low-risk youths was not seen for prior use of other tobacco products.

A prior model of the catalyst effects of e-cigarettes conceptualizes a 2-step process.^36^ In the first step, e-cigarettes attract low-risk youths to tobacco use through their flavors,^37^ perceived safety,^38^ and higher acceptance among peers.^39^ In the second step, e-cigarettes lead to the subsequent initiation of cigarettes, potentially through nicotine addiction,^37,40^ social and behavioral mechanisms including increased access and exposure to tobacco products or increased willingness to take risks,^36^ or through the development of a familiarity with the ritualistic procedures of smoking.^37^

Given that the PATH Study is a survey of a nationally representative cohort, the data permit at least a rough estimate of how many youths may be smoking cigarettes because they had first used e-cigarettes. When extrapolated to the population of US youths, our estimates suggest that as many as 178 850 youth ever smokers and 43 446 youth current smokers may have initiated combustible cigarettes through the e-cigarette pathway over the 2-year period between 2013 and 2014 and 2015 and 2016. The prevalence of past 30-day cigarette use among 10th graders was approximately 4.9% in 2016.^41^ Thus, our estimates suggest that this prevalence may have been 0.7% lower had there been no e-cigarette use by youths (4.9% × 15.3%). This estimate is lower than a prior estimate reporting that e-cigarettes would account for a 2% increase in smoking initiation rates under the hypothetical scenario where reported pooled ORs^18^ are accepted as causal.^42^

A recent trend analysis found that the decline in cigarette prevalence among youths accelerated by 2 to 4 times after 2014 when e-cigarette prevalence increased.^24^ Although the authors concluded the population-level catalyst effect of e-cigarettes is negligible,^24^ the accelerated rate of decline in smoking prevalence may instead reflect the success of recent tobacco control interventions.^24^ The projected 116 000 youths aged 11 to 18 years prevented from cigarette initiation annually by the Real Cost media campaign^43^ alone represent a larger decrease in cigarette use than the new cigarette use we estimate as attributable to e-cigarettes. Thus, the increase of e-cigarettes may have resulted in an increase in smoking prevalence under the true counterfactual scenario in which e-cigarettes entered a constant tobacco market. Additionally, our reverse analysis shows an association between cigarette smoking and subsequent e-cigarette use, suggesting that e-cigarettes may divert smokers toward e-cigarettes in some youths as well as in increasing risk of cigarette initiation in others. Nonetheless, the youths initiating cigarettes through e-cigarettes represent a substantial public health challenge that may warrant stricter regulation of youths’ access to e-cigarettes.

In the current study, we used a novel study design in which respondents’ reported tobacco product histories were used to establish the temporal ordering of tobacco and e-cigarette use. This information allowed us to capture product use transitions with increased granularity compared with studies relying on prospective measurement of product use transitions, reducing the potential for exposure misclassification that may occur when youths initiate products between waves. Furthermore, even though the inclusion criteria used in the present study were more restrictive than in prior studies, the focus on a cohort of youths who were completely tobacco naive at baseline is likely to enhance internal validity by circumventing the selective exclusion of e-cigarette users who had already progressed to cigarette use.

### Limitations

Despite its strengths, our study had several limitations. First, the PATH Study data are observational; we cannot establish causal relations or rule out the possibility of residual confounding by underlying risk-taking propensities. Second, our study design, which relied on recall to establish tobacco use timelines, did not allow us to examine how the association of e-cigarette use with cigarette initiation may vary by e-cigarette product characteristics or patterns of use because we did not use the more detailed questions on contemporaneous product use. Third, we included only individuals in each prior use category based on the first tobacco product they initiated. If e-cigarette use is implicated in transitions to smoking even in cases in which the product sequence, for example, was hookah to e-cigarettes to cigarettes, our findings may represent conservative estimates of the association between e-cigarette use and subsequent cigarette initiation. Our PAF calculations were based on an assumption of causality between exposure and outcome despite the aforementioned limitation of using observational data. Thus, our population-level estimates of the association of e-cigarette use with combustible cigarette initiation are provisional. Until additional evidence confirming these estimates is generated, they should be interpreted with caution.

## Conclusions

This large, nationally representative study of US youths supports the view that e-cigarettes represent a catalyst for cigarette initiation among youths. The association was especially pronounced in low-risk youths, raising concerns that e-cigarettes may renormalize smoking behaviors and erode decades of progress in reducing smoking among youths. Although the individual-level risk of cigarette initiation was comparable for prior e-cigarette users and prior other tobacco product users, the proportion of new cigarette use attributable to prior e-cigarette use appears larger than the proportion attributable to prior use of all other products combined. These findings strengthen the rationale for aggressive regulation of youth access to and marketing of e-cigarettes to achieve future decreases in the prevalence of cigarette use among youths.

## Notes

Supplement. **eTable 1. **Risk Group Stratification Criteria **eTable 2. **Sensitivity to Missing Data: Adjusted Probabilities and Odds of Cigarette Use at Wave 3 by Prior Tobacco Product Use Among 5079 Youths Aged 12-15 Years, Population Assessment of Tobacco and Health Study, 2013-2016 **eTable 3.** Sensitivity to Missing Data: Adjusted Probabilities and Odds of Cigarette Use at Wave 3 by Prior Tobacco Product Use among 5079 Youths Aged 12-15 Years, Stratified by Risk Group, Population Assessment of Tobacco and Health Study, 2013-2016 **eTable 4. **Sensitivity to OR Correction: Population-Level Proportion of Cigarette Use Attributable to Prior Use of E-Cigarettes and Other Tobacco Products Using Corrected Risk Ratios, Population Assessment of Tobacco and Health Study, 2013-2016 **eTable 5. **Reverse Analysis: Adjusted Probabilities and Odds of E-Cigarette Use at Wave 3 by Prior Tobacco Product Use Among 6123 Youths Aged 12-15 Years, Population Assessment of Tobacco and Health Study, 2013-2016 **eFigure. **Diagram of Inclusion/Exclusion Criteria
