# Supplementary material for: Association of Electronic Cigarette Use With Subsequent Initiation of Tobacco Cigarettes in US Youths
Source: JAMA Netw Open. 2019 Feb 1;2(2):e187794. doi: 10.1001/jamanetworkopen.2018.7794 (PMC6484602; doi:10.1001/jamanetworkopen.2018.7794)
Supplement: Supplement. — eTable 1. Risk Group Stratification Criteria eTable 2. Sensitivity to Missing Data: Adjusted Probabilities and Odds of Cigarette Use at Wave 3 by Prior Tobacco Product Use Among 5079 Youths Aged 12-15 Years, Population Assessment of Tobacco and Health Study, 2013-2016 eTable 3. Sensitivity to Missing Data: Adjusted Probabilities and Odds of Cigarette Use at Wave 3 by Prior Tobacco Product Use among 5079 Youths Aged 12-15 Years, Stratified by Risk Group, Population Assessment of Tobacco and Health Study, 2013-2016 eTable 4. Sensitivity to OR Correction: Population-Level Proportion of Cigarette Use Attributable to Prior Use of E-Cigarettes and Other Tobacco Products Using Corrected Risk Ratios, Population Assessment of Tobacco and Health Study, 2013-2016 eTable 5. Reverse Analysis: Adjusted Probabilities and Odds of E-Cigarette Use at Wave 3 by Prior Tobacco Product Use Among 6123 Youths Aged 12-15 Years, Population Assessment of Tobacco and Health Study, 2013-2016 eFigure. Diagram of Inclusion/Exclusion Criteria [file jamanetwopen-2-e187794-s001.pdf]

Supplementary Online Content

Berry KM, Fetterman JL, Benjamin EJ, et al. Association of electronic cigarette use with subsequent initiation of tobacco cigarettes in US youths. *JAMA Netw Open*. 2019;2(2):e187794. doi:10.1001/jamanetworkopen.2018.7794

eTable 1. Risk Group Stratification Criteria
eTable 2. Sensitivity to Missing Data: Adjusted Probabilities and Odds of Cigarette Use at Wave 3 by Prior Tobacco Product Use Among 5079 Youths Aged 12-15 Years, Population Assessment of Tobacco and Health Study, 2013-2016
eTable 3. Sensitivity to Missing Data: Adjusted Probabilities and Odds of Cigarette Use at Wave 3 by Prior Tobacco Product Use among 5079 Youths Aged 12-15 Years, Stratified by Risk Group, Population Assessment of Tobacco and Health Study, 2013-2016
eTable 4. Sensitivity to OR Correction: Population-Level Proportion of Cigarette Use Attributable to Prior Use of E-Cigarettes and Other Tobacco Products Using Corrected Risk Ratios, Population Assessment of Tobacco and Health Study, 2013-2016
eTable 5. Reverse Analysis: Adjusted Probabilities and Odds of E-Cigarette Use at Wave 3 by Prior Tobacco Product Use Among 6123 Youths Aged 12-15 Years, Population Assessment of Tobacco and Health Study, 2013-2016
eFigure. Diagram of Inclusion/Exclusion Criteria

This supplementary material has been provided by the authors to give readers additional information about their work.

© Berry KM et al. *JAMA Network Open.*

eTable 1. Risk Group Stratification Criteria

Category	Question	Check if true
Sensation-seeking personality traits	Strongly agree/agree with statement "I like to do frightening things"	☐
	Strongly agree/agree with statement "I like new and exciting experiences, even if I have to break the rules"	☐
	Strongly agree/agree with statement "I prefer friends who are exciting and unpredictable"	☐
Risk-taking behaviors	Ever used alcohol	☐
	Ever used marijuana	☐
	Ever used Ritalin, Adderall, painkillers, sedatives, or tranquilizers without a prescription	☐
Cigarette susceptibility	Very curious/somewhat curious/a little curious to question "Have you ever been curious about smoking a cigarette?"	☐
	Definitely yes/probably yes/probably not to question "Do you think you will smoke a cigarette in the next year?"	☐
	Definitely yes/probably yes/probably not to question "If one of your best friends were to offer you a cigarette, would you smoke it?"	☐
	**Any risk? =**	☐[a]

[a] Youths reporting any of the above statements/behaviors were categorized into the intermediate- and/or high-risk group. Youths reporting none of the above statements/behaviors were classified as low risk.

© Berry KM et al. *JAMA Network Open.*

eTable 2. Sensitivity to Missing Data: Adjusted Probabilities and Odds of Cigarette Use at Wave 3 by Prior Tobacco Product Use Among 5079 Youths Aged 12-15 Years, Population Assessment of Tobacco and Health Study, 2013-2016

	**Ever Cigarette Use**				**Current Cigarette Use**			
	**Adj. OR[b]**	**95% CI**	***P value***	**Adj. %[c]**	**Adj. OR[b]**	**95% CI**	***P value***	**Adj. %[c]**
**Prior tobacco product use[a]**								
No prior tobacco product use	1 [Ref]	NA	NA	4.5%	1 [Ref]	NA	NA	1.6%
Prior e-cigarette use	4.07	(2.88, 5.74)	<0.001	14.4%	2.26	(1.24, 4.12)	0.01	3.5%
Prior other product use	4.07	(2.71, 6.11)	<0.001	14.4%	3.55	(1.88, 6.73)	<0.001	5.3%

Abbreviations: e-cigarette, electronic cigarette; adj, adjusted; OR, odds ratio; Ref, reference; NA, not applicable.

[a] Youths were considered to have prior e-cigarette use if they started using e-cigarettes between wave 1 and wave 3 and their e-cigarette use preceded use of any other tobacco product. They were considered to have other product use if another non-cigarette tobacco product was their first product.

[b] Regression models and resulting probabilities were sample weighted and adjusted for sex, age, race and ethnicity, parental education, urban or rural residence, living with a tobacco user, noticing tobacco warnings, tobacco advertisement receptivity, and all nine individual measures of susceptibility/risk (ever alcohol use, ever marijuana use, prescription drug abuse, enjoying frightening things, liking new and exciting experiences, preferring unpredictable friends, willingness to smoke in next year, curiosity about cigarettes, and susceptibility to cigarette peer pressure from friends).

[c] Predicted probabilities were calculated via marginal standardization using coefficients estimated from regression models.

© Berry KM et al. *JAMA Network Open.*

eTable 3. Sensitivity to Missing Data: Adjusted Probabilities and Odds of Cigarette Use at Wave 3 by Prior Tobacco Product Use among 5079 Youths Aged 12-15 Years, Stratified by Risk Group, Population Assessment of Tobacco and Health Study, 2013-2016

	**Ever Cigarette Use**								
	**Intermediate- and/or High-Risk Group**				**Low-Risk Group**				***P* value for interaction**
	**n=3,000**				**n=2,079**				
	**Adj. OR[c]**	**95% CI**	***P* value**	**Adj. %[d]**	**Adj. OR[c]**	**95% CI**	***P* value**	**Adj. %[d]**	
**Prior tobacco product use[b]**									
No prior tobacco product use	1 [Ref]	NA	NA	6.8%	1 [Ref]	NA	NA	1.3%	NA
Prior e-cigarette use	3.51	(2.45, 5.02)	<0.001	18.7%	8.38	(3.42, 20.55)	<0.001	9.6%	0.07[e]
Prior other product use	3.96	(2.59, 6.06)	<0.001	20.4%	3.91	(1.14, 13.40)	0.03	4.8%	0.88[f]
	Current Cigarette Use								
**Prior tobacco product use[b]**									
No prior tobacco product use	1 [Ref]	NA	NA	2.5%	1 [Ref]	NA	NA	0.6%	NA
Prior e-cigarette use	1.75	(0.94, 3.28)	0.08	4.1%	11.20	(2.76, 45.44)	0.001	5.6%	0.04[e]
Prior other product use	3.56	(1.85, 6.85)	<0.001	7.8%	1.70	(0.12, 24.34)	0.70	1.0%	0.85[f]

Abbreviations: e-cigarette, electronic cigarette; adj, adjusted; OR, odds ratio; Ref, reference; NA, not applicable.

[a] Youths who reported ever alcohol use, ever marijuana use, prescription drug abuse, agreed with any of three statements on sensation seeking, or showed any susceptibility to any of the three questions on cigarette susceptibility were considered

© Berry KM et al. *JAMA Network Open.*

intermediate and/or high risk. Youths who had never used alcohol, never used marijuana, never abused prescription drugs, disagreed with all three sensation seeking statements, and showed no cigarette susceptibility were considered low risk.

[b] Youths were considered to have prior e-cigarette use if they started using e-cigarettes between wave 1 and wave 3 and their e-cigarette use preceded use of any other tobacco product. They were considered to have other product use if another non-cigarette tobacco product was their first product.

[c] Regression models and resulting probabilities were sample weighted and adjusted for sex, age, race and ethnicity, parental education, urban or rural residence, living with a tobacco user, noticing tobacco warnings, and tobacco advertisement receptivity. The intermediate- and/or high-risk strata was also adjusted for all nine individual measures of susceptibility/risk (ever alcohol use, ever marijuana use, prescription drug abuse, enjoying frightening things, liking new and exciting experiences, preferring unpredictable friends, willingness to smoke in next year, curiosity about cigarettes, and susceptibility to cigarette peer pressure from friends).

[d] Predicted probabilities were calculated via marginal standardization using coefficients estimated from regression models.

[e] P-value for interaction between prior e-cigarette use and being in the intermediate- and/or high-risk group.

[f] P-value for interaction between prior other product use and being in the intermediate- and/or high-risk group.

© Berry KM et al. *JAMA Network Open.*

eTable 4. Sensitivity to OR Correction: Population-Level Proportion of Cigarette Use Attributable to Prior Use of E-Cigarettes and Other Tobacco Products Using Corrected Risk Ratios, Population Assessment of Tobacco and Health Study, 2013-2016

	**Ever Cigarette Use**				**Current Cigarette Use**			
	**RR**	**PAF**	**Total New Users over 2 Years**	**Attributable Users**	**RR**	**PAF**	**Total New Users over 2 Years**	**Attributable Users**
Prior e-cigarette use	3.66[a]	20.9%[b]	820,414	171,467[c]	2.68[d]	15.1%	283,964	42,879[f]
Prior other product use	3.46[g]	12.3%[h]	820,414	100,911[i]	3.32[j]	13.6%[k]	283,964	38,619[l]
**Overall**		33.2%	820,414	272,378		28.7%	283,964	81,498

Abbreviations: e-cigarette, electronic cigarette; RR, risk ratio; OR, odds ratio; PAF, population attributable fraction

[a] RR = (OR for prior e-cigarette use)/[(1 - incidence of ever cigarette use among youth with no prior tobacco use) + (incidence of ever cigarette use among youth with no prior tobacco use x OR for prior e-cigarette use)] = (4.09)/[(1-0.0382)+(0.0382 x 4.09)] = 3.66

[b] PAF= [proportion of ever cigarette users with prior e-cigarette use (RR for prior e-cigarette use – 1)] / (RR for prior e-cigarette use) = [.288(3.66-1)]/(3.66)=20.9%

[c] Attributable users = (PAF)(Total New Users) = (0.209)(820,414) = 171,467

[d] RR = (OR for prior e-cigarette use)/[(1 - incidence of current cigarette use among youth with no prior tobacco use) + (incidence of current cigarette use among youth with no prior tobacco use x OR for prior e-cigarette use)] = (2.75)/[(1-0.0139)+(0.0139 x 2.75)] = 2.68

[e] PAF= [proportion of current cigarette users with prior e-cigarette use (RR for prior e-cigarette use – 1)] / (RR for prior e-cigarette use) = [.241(2.68-1)]/(2.68)=15.1%

[f] Attributable users = (PAF)(Total New Users) = (0.151)(283,964) = 42,879

[g] RR = (OR for prior other product use)/[(1 - incidence of ever cigarette use among youth with no prior tobacco use) + (incidence of ever cigarette use among youth with no prior tobacco use x OR for prior other product use)] = (3.84)/[(1-0.0382)+(0.0382 x 3.84)] = 3.46

[h] PAF= [proportion of ever cigarette use with prior other product use (RR for prior other product use – 1)] / (RR for prior other product use) = [.173(3.46-1)]/(3.46)=12.3%

[i] Attributable users = (PAF)(Total New Users) = (0.123)(820,414) = 100,911

© Berry KM et al. *JAMA Network Open.*

[j] RR = (OR for prior other product use)/[(1 - incidence of current cigarette use among youth with no prior tobacco use) + (incidence of current cigarette use among youth with no prior tobacco use x OR for prior other product use)] = (3.43)/[(1-0.0139)+(0.0139 x 3.43)] = 3.32

[k] PAF= [proportion of current cigarette use with prior other product use (RR for prior other product use – 1)] / (RR for prior other product use) = [.194(3.32-1)]/(3.32)=13.6%

[l] Attributable users = (PAF)(Total New Users) = (0.136)(283,964) = 38,619

© Berry KM et al. *JAMA Network Open.*

eTable 5. Reverse Analysis: Adjusted Probabilities and Odds of E-Cigarette Use at Wave 3 by Prior Tobacco Product Use Among 6123 Youths Aged 12-15 Years, Population Assessment of Tobacco and Health Study, 2013-2016

	**Ever E-Cigarette Use**				**Current E-Cigarette Use**			
	**Adj. OR[b]**	**95% CI**	***P value***	**Adj. %[c]**	**Adj. OR[b]**	**95% CI**	***P value***	**Adj. %[c]**
**Prior tobacco product use[a]**								
No prior tobacco product use	1 [Ref]	NA	NA	9.9%	1 [Ref]	NA	NA	2.5%
Prior cigarette use	3.51	(2.40, 5.14)	<0.001	25.4%	3.84	(2.10, 7.05)	<0.001	8.3%
Prior other product use	1.81	(1.28, 2.54)	0.001	15.8%	2.47	(1.42, 4.29)	0.001	5.7%

Abbreviations: e-cigarette, electronic cigarette; adj, adjusted; OR, odds ratio; Ref, reference; NA, not applicable.

[a] Youths were considered to have prior cigarette use if they started using cigarettes between wave 1 and wave 3 and their cigarette use preceded use of any other tobacco product. They were considered to have other product use if a non-cigarette tobacco product besides an e-cigarette was their first product.

[b] Regression models and resulting probabilities were sample weighted and adjusted for sex, age, race and ethnicity, parental education, urban or rural residence, living with a tobacco user, noticing tobacco warnings, tobacco advertisement receptivity, and all nine individual measures of susceptibility/risk (ever alcohol use, ever marijuana use, prescription drug abuse, enjoying frightening things, liking new and exciting experiences, preferring unpredictable friends, willingness to smoke in next year, curiosity about cigarettes, and susceptibility to cigarette peer pressure from friends).

[c] Predicted probabilities were calculated via marginal standardization using coefficients estimated from regression models.

© Berry KM et al. *JAMA Network Open.*

eFigure. Diagram of Inclusion/Exclusion Criteria

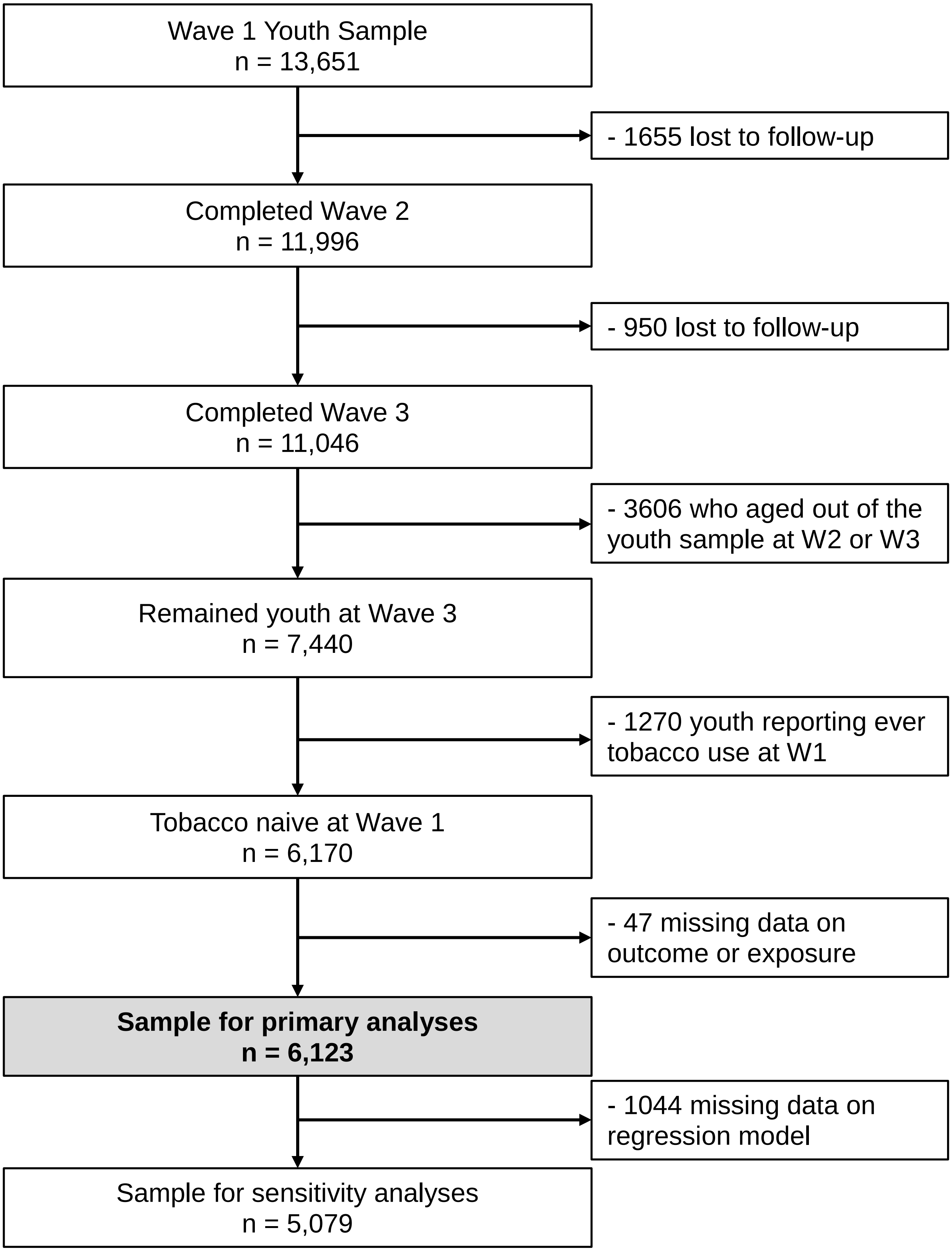

© Berry KM et al. *JAMA Network Open.*
